# EIP on AHA Ontology for adherence: Knowledge representation advanced tools

**Published:** 2019-01-06

**Authors:** E Román-Villarán, FP Pérez-Leon, GA Escobar-Rodriguez, CL Parra-Calderón

**Affiliations:** 1 Biomedical Informatics, Biomedical Engineering and Health Economy R&I Group. Institute of Biomedicine of Seville, IBiS/”Virgen del Rocío” University Hospital/CSIC/University of Seville, Seville, Spain; 2 Head of Innovation Technology, “Virgen del Rocío” University Hospital, Seville, Spain

**Keywords:** Adherence, Knowledge, polypharmacy, chronic diseases, older patients

## Abstract

Nowadays diseases tend to chronicle, mainly due to the increase in life expectancy and this leads to a state of polypharmacy. More than 1.5% of Spain’s GDP is spent on pharmaceuticals and healthcare products. Complex chronic patients (pluripathological and polymedicated) account for most of the expenditure. The “Action Group A1” of the European Innovation Partnership develops in the “Active and Healthy Ageing” programme actions to improve the quality of life and health outcomes of these patients. On the other hand, the PITeS TIiSS project develops decision support tools to improve this scenario. An ontology has been developed as a tool on adherence. The domain of this ontology is mainly focused on medication adherence and measurement methods. This ontology gathers the necessary knowledge about the domain allowing the use of the ontology as part for is possible.

## I. INTRODUCTION

Adherence to medication defines as the process by which the patients take their medication as prescribed [1] and is divided into three phases: initiation, implementation, and interruption.

Today, there is an increase in life expectancy. This causes an older population. This situation is related to an increase in multimorbidities and chronic diseases, leading to a state of polypharmacy [2].

There are studies such as Onder et al. [3] that evaluate the prescribing patterns in older patients. They conclude that male patients are prescribed more doses and those female patients are much more sensitive to medications.

Only 50% of patients with chronic diseases have adherence to treatment [4].

More than 1,5% of the Spanish GDP is spent on pharmaceuticals and healthcare products. Complex chronic patients (pluripathological and polymedicated) account for most of the expenditure. The “Action Group A1” of the “European Innovation Partnership on Active and Healthy Ageing” is working to improve adherence to therapeutic plans for pluripathological patients and other chronically ill patients [5].

There are many studies to improve adherence. Some of these studies propose tools that monitor medication may be the one proposed by Hale et al. [6] called MedSentry. They found that adherence was greatly improved in patients with complex heart failure treatments. It also reduced the number of hospitalizations and the use of the emergency system. Karanasiou et al. [7] propose a tool for predicting patient adherence based on the HEARTEN project.

In the sphere of mobile apps, there is a proposal by Davies et al. [8], for more exhaustive monitoring. They concluded that the adhesion depends very much on the daily routine.

In addition, several studies have been done such as Marchionni et al. [9] in reference to the adherence of serotonin reuptake inhibitors in older patients. They concluded that patients who are adherents to this type of medications are more adherents to other medications with the exception of anticoagulants. Wuyts et al. [10] did a study on treatment adherence in Belgium primary care. This helped to increase adherence as it made it possible to detect the problems for which treatment adherence does not occur and thus to find solutions to them.

The SYMPATHY project, proposed by McIntosh et al. [2], believe it’s necessary to improve the efficiency in the health system and to prepare healthcare professionals for a good approach to population aging. Also in this area is the EMERGE guide presented by Grounded et al. [11] to improve the adherence.

Zaugg et al. [12] reviewed nine studies in this area and concluded that if healthcare professionals were informed about their patients’ actual compliance, adherence would be significantly improved.

With respect to the most commonly used tools for adherence control, Giardini et al. [13] describe their advantages and disadvantages.

In recent years, the ontologies development (explicit formal specifications of terms in a knowledge domain and their relationships [14]) has moved from the realm of Artificial Intelligence laboratories to that of the desks of domain experts. Many disciplines are now developing standardized ontologies that subject matter experts can use to share, enrich and annotate information in their fields. In biomedicine, the best examples are the large standardized and structured vocabularies such as SNOMED [15] and the semantic network of the Unified Medical Language System (UMLS) [16].

In the adherence domain, there are important taxonomy definition works such as the project experience “Ascertaining Barriers to Compliance” (ABC) [1]. Even a specific computing experience to predict the adherence level based on ABC concepts and applying the ISPOR recommendations [17]. Although in this case the models are based on conventional statistics, they are sophisticated models.

The biomedicine and healthcare have already been demonstrated that the use of ontologies has a high level of maturity for the knowledge representation and management such as the artificial intelligence application pillar in these domains [18].

## II. METHODOLOGY

Ontologies, therefore, can help to knowledge management. Their uses are very varied:

- They are used as vocabulary sources to annotate data or index documents, which serves primarily for accurate retrieval.
- Integrate heterogeneous data from disparate sources (essential for translational research)
- Study definition groups and their characteristics.
- Help standardize and integrate data sources.
- They are computable domain knowledge sources.
- Help in language processing [19].

The adherence ontology proposed in the PITeS TIiSS project (1) defines adherence and its phases by alluding to persistence and its measurement, (2) develops which factors influence the patient adherence degree, (3) includes methods and indicators used and validated by the scientific community to measure the therapeutic compliance of a patient and (4) allows the classification of the adherence level of a patient based on the methods collected.

The objective for which this ontology was constructed was to gather the knowledge about the therapeutic regimen adherence of complex patients and the existing validated methods to know the accuracy degree to the prescribed treatment of each patient. The reason why it has been decided to use ontology is that it uses a language easily interpretable by the human-machine system, which also allows inference between concepts.

In order to create the ontology, a bibliographic review of scientific articles on adherence to medication has been carried out, as well as the methods for its measurement. This review has been done constantly to keep always updated the information entered. The free software “Protégé” has been used as editor of the ontology. This has a simple interface and allows to generate the ontology in Web Ontology Language (.OWL) or other forms of representation automatically. Concepts related to patient context factors that influence adherence have been strategically included in a negative way so that it is possible to deduce that the higher the number of factors, the lower the degree of adherence.

The strategy for capturing concepts and proposing a coherent hierarchy has been based on defining the types of adherence. Within the adherence to medication, influential factors, adherence phases to mediation and indicators have been defined, as well as types of indicators. In each of these concepts, it has continued to expand with concepts associated. It is in the section on indicators that the logic of the results that the indicators can give has been introduced. In order to have traceability of the concepts introduced, the reference articles have been incorporated as annotations of each concept.

Once the concepts have been obtained, they have been mapped with SNOMED CT and the code has been added in the ontology. Thanks to this, we have been able to identify 66 of the concepts of the proposed ontology with SNOMED CT codes. In addition, the concepts have been mapped with FHIR terminology in a tabulated document.

Other elements that complete the proposed ontology are concepts definitions with the Royal Academy of Medicine dictionary.

In addition to scientific articles, clinical guidelines such as the PAI of pluripathological patients [20] and the Personalised Action Plan in pluripathological patients or patients with complex health needs [21] have been revised.

## III. RESULTS

**Fig. 1:**
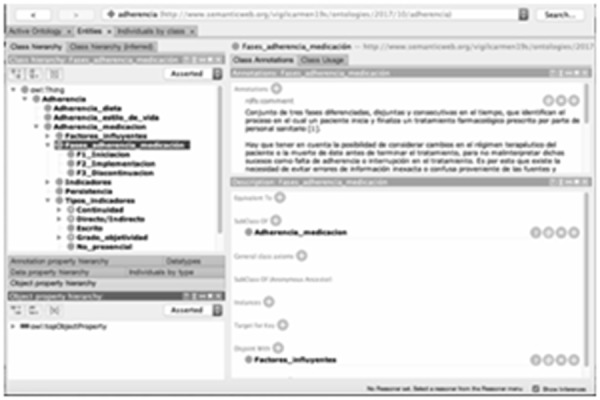
Adherence ontology

The ontology has been created by collecting relevant information on the concept of adherence (adherence to diet, lifestyle, and medication), developing an adherence to medication including its phases, as well as factors pertaining to the patient’s context (patient conditions, health system, socio-demographic, therapeutic, etc.) and illness that may influence the level of adherence to medical treatment (biological). In the current version of the ontology 1096 axioms, 121 classes and 89 members have been included (the members are characteristics within each class and subclass that complete the information in a more detailed way). After reviewing a large number of references, 86 of them have been introduced to complete this ontology (Figure 1).

In addition, it has been classified according to their inherent characteristics those existing methods that are considered validated by the scientific community to assess the patient’s degree of adherence, including the concepts necessary for the application of the methods.

The developed ontology is capable of classifying the patient adherence degree from the input information corresponding to the result obtained by the patient after completing one of the collected adherence assessment methods.

## IV. DISCUSSION

Ontologies are tools with a series of characteristics that make them very useful:

- They provide definitions of terms.
- They provide a set of labels associated with the classes and relationships in the ontology.
- Its main components are classes and relationships.
- They provide machine-readable axioms and formal definitions.

Thanks to these features, many tools and methods make use of ontologies such as data annotation and integration, vocabularies, formalized definition and axioms, and multimodal data analysis [22].

An example of the use of ontology is that proposed by Bandrowski et al. [23]. It provides terms with precisely defined meanings to describe all aspects of biological and medical research. They cover the whole process of planning, execution, and reporting.

One of the major problems affecting patient adherence is also the lack of clarity in terminology. This issue leads to inconsistencies and disagreements between publications and healthcare professionals and further decreases communication between doctor and patient. This situation occurs above all with the terms compliance, adherence, persistence and concordance [24].

In the field of decision support tools is the one proposed by Galopin et al. [25]. They justify that, with ontological reasoning, the tool works much better. In this way, it is possible to model several Clinical Practice Guides at the same time and relate all concepts to the ontology. On the other hand, Zhang et al. [26] propose a tool to support clinical decision making during follow-up evaluations of patients with chronic diseases at home. They use the ontology to enter patient data, medical knowledge, and evaluation criteria.

In this sense and as future work, we intend to use ontology as part of a semantic infrastructure that will be combined with other automatic learning methods, decision rules, prediction and classification, and other Artificial Intelligence algorithms. All these components will form a System to Support the Personalized Clinical Decision to Complex Chronic Patients.

## V. CONCLUSION

Lack of adherence to medication is one of the significant health problems. It affects the health of patients, the work and outcomes of health professionals and the economic sphere. This consequence is why it must be faced from all possible scenarios to try to reduce this problem.

A negative situation affecting 50% of patients should be studied and analyzed intensively.

Ontologies are useful tools for identifying the elements involved in patient adherence from all points of view. This allows to obtain a lot of information in a very short time and well organized.

A system has been constructed that brings together the main factors that make it possible to understand the degree of adherence that a patient has. Knowing these factors allows the health professional to act accordingly in search of improvement in adherence. It is also a tool capable of classifying the level of adherence of a patient on the basis of the common methods of evaluation of adherence used in clinical practice. This allows it to be implemented in a clinical decision support system as well as semantic interoperability between systems.

## References

[1] Vrijens B, De Geest S, Hughes DA, Przemyslaw K, Demonceau J, Ruppar T (2012). A new taxonomy for describing and defining adherence to medications. British journal of clinical pharmacology.

[2] McIntosh J, Alonso A, MacLure K, Stewart D, Kempen T, Mair A, Gennimata D (2018). A case study of polypharmacy management in nine European countries: Implications for change management and implementation. PloS one.

[3] Onder G, Marengoni A, Russo P, Degli Esposti L, Fini M, Monaco A (2016). Advanced age and medication prescription: more years, less medications? A nationwide report from the Italian Medicines Agency. Journal of the American Medical Directors Association.

[4] Papadopoulos H, Giardini A, Costa E, Monaco A, Mair A, Cena C (2016). Increasing adherence in therapies and polypharmacy in Europe: EIP on AHA Action Group A1 activities toward integrated care information systems and self-management applications. Digital Medicine.

[5] European Innovation Partnership on Active and Healthy Ageing (2016). A1 ACTION GROUP 2016–2018. Version 9th.

[6] Hale TM, Jethwani K, Kandola MS, Saldana F, Kvedar JC (2016). A remote medication monitoring system for chronic heart failure patients to reduce readmissions: a two-arm randomized pilot study. Journal of medical Internet research.

[7] Karanasiou GS, Tripoliti EE, Papadopoulos TG, Kalatzis FG, Goletsis Y, Naka KK (2016). Predicting adherence of patients with HF through machine learning techniques. Healthcare technology letters.

[8] Davies MJ, Kotadia A, Mughal H, Hannan A, Alqarni H (2015). The attitudes of pharmacists, students and the general public on mHealth applications for medication adherence. Pharmacy practice.

[9] Marengoni A, Onder G, Degli Esposti L, Russo P, Sangiorgi D, Buda S, Fini M, Marchionni N, Bonassi S, Mammarella F, Marrocco W, Pozzi G, Palmer K, Monaco A, Pecorelli S, Pani L, Geriatrics Steering Committee of the Italian Medicines Agency on behalf of the OsMed Health-DB Network (2016). Adherence to Selective Serotonin and Serotonin-Norepinephrine Reuptake Inhibitor Prescriptions Affects Overall Medication Adherence in Older Persons. J Clin Psychiatry.

[10] Wuyts J, Maesschalck J, De Wulf I, Foubert K, Boussery K, De Lepeleire J (2018). Studying the impact of a medication use evaluation for polymedicated older patients by the community pharmacist (SIMENON): study protocol. BMC health services research.

[11] Grounded ARGE (2018). ESPACOMP Medication Adherence Reporting Guideline (EMERGE). Ann Intern Med.

[12] Zaugg V, Korb-Savoldelli V, Durieux P, Sabatier B (2018). Providing physicians with feedback on medication adherence for people with chronic diseases taking long-term medication. The Cochrane Library.

[13] Giardini A, Martin MT, Cahir C, Lehane E, Menditto E, Strano M (2016). Toward appropriate criteria in medication adherence assessment in older persons: position paper. Aging clinical and experimental research.

[14] Gruber TR (1993). A translation approach to portable ontology specifications. Knowledge acquisition.

[15] Stearns MQ, Price C, Spackman KA, Wang AY (2001). SNOMED clinical terms: overview of the development process and project status.

[16] Lindberg DA, Humphreys BL, McCray AT (1993). The unified medical language system. Yearbook of Medical Informatics.

[17] Hutchins DS, Zeber JE, Roberts CS, Williams AF, Manias E, Peterson AM (2015). Initial medication adherence—review and recommendations for good practices in outcomes research: an ISPOR Medication Adherence and Persistence Special Interest Group report. Value in Health.

[18] Dhombres F, Charlet J (2018). As Ontologies Reach Maturity, Artificial Intelligence Starts Being Fully Efficient: Findings from the Section on Knowledge Representation and Management for the Yearbook 2018. Yearbook of medical informatics.

[19] Bodenreider O (2008). Biomedical ontologies in action: role in knowledge management, data integration and decision support. Yearbook of medical informatics.

[20] Ollero Baturone M, Bernabeu-Wittel M, Espinosa Almendro JM, García Estepa R, Morilla Herrera JC, Pascual de la Pisa B (2018). Atención a pacientes pluripatológicos: proceso asistencial integrado.

[21] Ollero Baturone M (2016). Plan de acción personalizado en pacientes pluripatológicos o con necesidades complejas de salud. Escuela Andaluza de Salud Pública.

[22] Hoehndorf R, Schofield PN, Gkoutos GV (2015). The role of ontologies in biological and biomedical research: a functional perspective. Briefings in bioinformatics.

[23] Bandrowski A, Brinkman R, Brochhausen M, Brush MH, Bug B, Chibucos MC (2016). The ontology for biomedical investigations. PloS one.

[24] Ahmed R, Aslani P (2014). What is patient adherence? A terminology overview. International journal of clinical pharmacy.

[25] Galopin A, Bouaud J, Pereira S, Séroussi B (2015). An Ontology-Based Clinical Decision Support System for the Management of Patients with Multiple Chronic Disorders. MedInfo.

[26] Zhang YF, Gou L, Zhou TS, Lin DN, Zheng J, Li Y, Li JS (2017). An ontology-based approach to patient follow-up assessment for continuous and personalized chronic disease management. Journal of biomedical informatics.

